# Military Hospital Construction

**Published:** 1916-11-04

**Authors:** S. Perkins Pick


### MILITARY HOSPITAL CONSTRUCTION.

By S. Perkins Pick, F.R.I.B.A.

When, in the autumn of 1914, the war cloud burst over Europe and Britain became involved in a sanguinary struggle of unparalleled magnitude, the nation was suddenly confronted with many complicated problems which demanded immediate solution. There was no time for leisurely discussion of details; something practical had to be done, and it was vital to set about it promptly. By no means the least bewildering was the problem of securing adequate medical treatment and hospital accommodation for the casualties that would inevitably occur from the very commencement of hostilities, and the way in which this was handled reflects the greatest credit upon all concerned, from Sir Alfred Keogh, Director-General of the Army Medical Service, to the humblest supernumerary. To find the buildings immediately required was no light matter, but the provision of efficient staffs of doctors and nurses no doubt presented even greater difficulties. For it must be remembered that the country was wholly unaccustomed to the idea of war on a large scale, and even educated people were quite unable to realise the necessity for all the hospitals which were springing up around them in a manner that suggested—to many minds—an excess of military zeal rather than a discerning effort to meet a most urgent need. We are all wiser now and better able to appreciate the foresight which initiated a series of humane activities that have become the heroic commonplaces of a tragic time.

### The Conversion of Existing Buildings.

In those early days—before people had learnt what bitter experience has since taught them—one frequently heard it said that to build hospitals in the Midlands and the North, away from the coast and remote from the theatre of war, was an insane waste of public money; that all these buildings were not required and many of them would never be occupied. Well, these facile optimists have had to change their tune, and they ought to be thankful that those in authority had a clearer vision of future possibilities. Some of these inland hospitals have already had as many as 30,000 patients through their doors, and the total number of beds within the United Kingdom may fairly be estimated at over 200,000. Schools, colleges, and factories were quickly turned into hospitals, and many large private houses were placed by their owners at the disposal of the Army Medical Service. The utility of these hastily adapted buildings varied considerably, but it was through their being so promptly available, and because nearly all the great hospitals supported by voluntary contributions came to the assistance of the War Department, that the early cases received proper attention. Poor-Law infirmaries and other public buildings were also made use of, and help of the utmost value was given by those responsible for the numerous mental hospitals up and down the country. The Board of Control divided the kingdom into districts, and by systematically emptying at least one asylum in each and transferring the patients to every kind of room that was at all suitable, they were able to provide good accommodation for several thousand military beds. This was a service of extreme value, rendered at a time when the difficulty was most acute, and great is our debt to those who so skilfully made the necessary adjustments. In addition to these well-directed efforts, many new hospitals of a temporary character were put up, and there were many extensions and alterations of existing buildings which had been taken over by the authorities. Some of these institutions were more costly than others, and all were not equally useful, but the experience gained in creating them should prove of the utmost value, not only for war-time, but in helping us to determine the most suitable arrangement for general hospitals.

### Base and Subsidiary Hospitals.

For war purposes perhaps the best scheme yet evolved is the one by which 50 per cent. of the patients are accommodated in a large and fully equipped base hospital, which should be near a good-sized town, and the remaining 50 per cent. distributed in a number of smaller buildings within easy reach of the base. Taking as a standard 3,000 beds, this arrangement places 1,500 patients—these should be the more serious cases—at the base, and 1,500 in the twenty or thirty smaller hospitals grouped round it. It is essential that the base should be provided with every modern resource, and, in addition to the wards, there must be operating theatres, an *x*-ray department, kitchens and stores for linen, clothing, groceries, and other necessary articles. Spacious administration offices are required for the base and also for the smaller hospitals associated with it. A well-fitted dispensary is essential, and there should be a pack store fitted with pigeon-holes for the patients' kits, with separate rooms for receiving, disinfecting, and mending these. It is very desirable to have a large dining-room adjacent to the kitchen. Many of the men are able to leave the wards, and it is both good for them and a saving of labour that they should take their meals in a central dining-room. Small observation wards are necessary, and these may adjoin the main wards. Isolation wards have been required, but not to any great extent. This is somewhat surprising, and a partial explanation may lie in the fact that special hospitals for certain cases have been provided. Wards for electrical treatment and massage are extremely useful, for where these have been supplied great benefit has been derived from the various devices for exercising limbs, and from frictions and vibrations. In most cases these valuable adjuncts have been supplied by gift. Pathological laboratories have been most useful in large hospitals and are essential to accurate diagnosis. A proper mortuary is indispensable.

The motor transport arrangements have often been undertaken by voluntary helpers, who have made great sacrifices in carrying out this useful work. The prompt and careful removal of wounded men from hospital trains to the base hospitals, the transference of patients from base to auxiliary hospitals, and the conveyance of soldiers discharged as fit from hospitals to railway stations, have been accomplished efficiently and with the greatest good will. In some cases the necessary ambulances have been garaged in the town near the base, in others sheds have been erected for them at the hospitals. The latter is undoubtedly the better arrangement, and it is also desirable that accommodation should be provided for staff motor cars. It is not reasonable that these cars should be left out of doors—sometimes for hours at a time—in all weathers.

With regard to laundry work, in the case of large hospitals it is no doubt convenient that this should be done on the premises; but this is not essential, for where the washing has had to be sent out to steam laundries no great difficulty has been experienced.

### Necessity for Recreation Facilities.

The provision of suitable games and diversions for the men is a matter of great importance. A good recreation hall, which can also be used for religious services, is an immense boon to a large hospital. It is rather surprising that the War Office does not sanction expenditure upon such buildings, considering the excellent use made of them where they have existed. A dining-room is not sufficient, for the constant clearing and resetting of tables, and the smell of cooking, render it unsuitable for purposes of recreation. Cricket and football grounds are most desirable, and provision for croquet, clock golf, and similar games should be made whenever possible. It is a real pleasure to see convalescent men enjoying such pastimes, and no doubt healthy amusement—especially in the open air—facilitates recovery and is a wholesome tonic. It should also not be forgotten that reasonable facilities for recreation are good for medical staffs and orderlies. While the gardens of some hospitals have been sorely neglected, those of others have been well cared for, often by the patients themselves. Pleasant surroundings have a salutary effect upon the nerves, and nothing could be better for a war-worn soldier than to find peaceful occupation in a garden. The amenities of convalescence deserve the most careful consideration, and the general effort that has been made to enhance them in the case of our gallant wounded is most commendable.

Possibly none of the buildings taken over by the War Office were able to satisfy all the requirements enumerated above. The asylums would in many cases be the most suitable, for they all have good kitchens and ample space for recreation. Their drawback is that they do not possess pack stores or sufficient operation theatres, *x*-ray apparatus, nurses' quarters, and other essentials of a military hospital. Supplying these deficiencies and carrying out necessary internal alterations have involved great expenditure, which must in some cases have amounted to about £20 per bed, exclusive of the cost of reinstatement. Hundreds of doors to single rooms had to be removed, staircases had to be widened for the passage of stretchers, and windows had to be made to open wide. Such adjustments are bound to cost money; but, in spite of the inevitable outlay, these commodious buildings have proved an exceedingly valuable asset. Most of them possess good engineering plants, which greatly facilitate the working of an institution where steam is required for cooking, laundry work, heating, electric lighting, etc. Many of the Poor-Law infirmaries taken over had excellent wards, good grounds, and complete administration offices, and the cost of adapting them was relatively small. But in some instances the Guardians were obliged to retain a portion of the buildings for the paupers in their charge, and there are obvious objections—both practical and sentimental—to such dual arrangements. However, the Poor-Law authorities did what they could, and this amounted to a national service that should be gratefully acknowledged. Asylums and Poor-Law infirmaries have provided excellent bases. Round these large central buildings many auxiliary hospitals have been grouped, and thus the number of available beds has been enormously increased.

School and college buildings have been chiefly useful as subordinate hospitals, for they have not the administration offices necessary to a base, and they are often so surrounded by population as to make their extension impracticable. They have no accommodation for nurses and orderlies, and their asphalted playgrounds are not ideal resorts for afflicted men. They have been useful in providing additional beds for less serious cases; but, where they have been mainly relied upon, one hardly sees how a satisfactory and economical arrangement could ensue. The same may be said of the factories and other miscellaneous buildings that have been utilised so extensively. But one advantage of the highest moment has been gained by the conversion of these various places into military hospitals of one sort and another. Throughout the land the presence of our wounded heroes in their familiar uniform has brought home to people the gravity of the times. Voluntary help has been stimulated, and a righteous animosity against the brutally aggressive Power which has forced this suffering upon the world has been kept alive. The sacrifices made by the Empire's youth have been vividly realised, the national spirit has been aroused, and —with a few contemptible exceptions—men and women have united in the stern resolve that these sacrifices shall not have been in vain. Bearing all this in mind, we will say no more of technical deficiencies and economic drawbacks.

### Temporary Hospital Construction.

We will now briefly consider the temporary hospitals which have been erected. The suddenness with which the war came upon us made it impossible for the War Department alone to deal with the huge amount of work that it entailed, and many outside architects were employed upon military hospitals. It is surprising that no attempt seems to have been made to secure the services of well-known men with special knowledge; indeed, the selection was apparently governed by chance. This is much to be regretted, for however capable an architect may be in other ways, it is obvious that those who have had special experience—and have learnt what to avoid—are in a better position to improve upon what has been done in the past than are men—however talented and conscientious—whose activities have been in other directions. However, in spite of this omission to choose the best available men, much good work has been done and valuable experience has been gained. At the beginning the model plans issued by the War Office provided for standard wards, each containing about twenty beds only. The cost of a hospital of this type could not have worked out at much less than £80 per bed. Outside architects soon showed that wards of much larger size were not only practicable but eminently desirable, and a number of hospitals were built with wards for at least fifty beds. By this means the number of administrative rooms was reduced by about one-half, and economy was effected in the sanitation department. Moreover, the cost was probably not 50 per cent. of that necessitated by the War Office scheme. In other cases expense was still further brought down by the institution of open-air wards—which allowed the beds to be placed closer together—and by reducing the quality of construction. By such contrivances the cost was brought down to about £20 per bed. The Government is indebted to those who helped in this evolutionary process, and quickly availed itself of the knowledge gained from it. The recent order that future buildings should be designed and carried out by the Government staff exclusively is therefore somewhat amazing. One of the latest plans executed by a Government department adopts the unit of 100 beds in a ward 150 feet long, 40 feet wide, and only 10 feet high. This means four rows of beds with twenty-five in each, with 60 feet superficial space and 600 feet cubic space for each bed. Such figures are the more surprising when it is realised that these most recent wards are enclosed and have a rather contracted window area. The only plausible reasons that can be given for an arrangement of this character are that in a base hospital the wards are not all occupied for many days together, and that the weeding-out of slightly wounded patients, who are transferred expeditiously to auxiliary hospitals, renders it unnecessary to provide as much superficial and cubic space as would be called for in a general hospital where all the beds are continuously occupied. It would, however, be interesting to know what is thought of these wards by those who have the working of them.

The open-air wards that have been established appear to have justified themselves. Many surgeons and nurses who have had experience both of closed and open wards strongly incline to the latter. It is claimed for them that wounds heal more quickly and that they promote the general health of patients. In very cold weather, nurses—whose arms are constantly being immersed in water—must find them very trying; but even among them we believe a consensus of opinion favours the open-air principle. Yet we find the Government adopting the 100-bed enclosed wards described above. This is certainly considered a backward movement by many hospital authorities, and we should like to learn, in due course, what is the justification of the official policy. It is to the public advantage that these matters should be fully discussed and impartially considered. Most valuable of all will be the opinions of doctors and nurses who have had to manage a variety of wards in one hospital. As soon as the strain of war work is over we hope some of these ladies and gentlemen will give the readers of this journal the benefit of their experience, and enlighten them as to the conclusions this experience has brought them to.

Reviewing the whole question in the light of two years' experimental knowledge, we believe a strong case can be made out for the erection of new buildings on open sites, limiting the number of patients to not more than eighty to the acre. Frequently the buildings commandeered have been a hindrance to the making of a good hospital; and it is very doubtful whether new buildings would have cost as much money, or occupied as much time in their erection, as was expended in making the necessary alterations to buildings already in existence. This criticism applies to base hospitals only. In the case of auxiliary hospitals, where the requirements are simpler, it is obviously cheaper and better to alter an existing building, of approximate suitability, than to put up a new one.

War has thrown many things into the melting-pot, and great changes are bound to come over the hospital world. If doctors and nurses and architects will pool the knowledge that military work has given them, such knowledge—rightly used when the time of reconstruction arrives—should greatly benefit humanity during the long years of peace that we trust are before us.

